# Lysophosphatidic Acid Increases the Electrophysiological Instability of Adult Rabbit Ventricular Myocardium by Augmenting L-Type Calcium Current

**DOI:** 10.1371/journal.pone.0045862

**Published:** 2012-09-21

**Authors:** Yong Wei, Li-qun Zhao, Bao-zhen Qi, Xing Xiao, Li He, Gen-qing Zhou, Song-wen Chen, Hong-li Li, Lei Ruan, Cun-tai Zhang, Shao-wen Liu

**Affiliations:** 1 Department of Cardiology, Shanghai First People’s Hospital, School of Medicine, Shanghai Jiao Tong University, Shanghai, China; 2 Department of Cardiology, Shanghai Songjiang Center Hospital, Shanghai, China; 3 Department of Geratology, Tongji Hospital, Tongji Medical College, Huazhong University of Science and Technology, Wuhan, China; Cinvestav-IPN, Mexico

## Abstract

Lysophosphatidic acid (LPA) has diverse actions on the cardiovascular system and is widely reported to modulate multiple ion currents in some cell types. However, little is known about its electrophysiological effects on cardiac myocytes. This study investigated whether LPA has electrophysiological effects on isolated rabbit myocardial preparations. The results indicate that LPA prolongs action potential duration at 90% repolarization (APD_90_) in a concentration- and frequency-dependent manner in isolated rabbit ventricular myocytes. The application of extracellular LPA significantly increases the coefficient of APD_90_ variability. LPA increased L-type calcium current (I_Ca,L_) density without altering its activation or deactivation properties. In contrast, LPA has no effect on two other ventricular repolarizing currents, the transient outward potassium current (I_to_) and the delayed rectifier potassium current (I_K_). In arterially perfused rabbit left ventricular wedge preparations, the monophasic action potential duration, QT interval, and Tpeak-end are prolonged by LPA. LPA treatment also significantly increases the incidence of ventricular tachycardia induced by S_1_S_2_ stimulation. Notably, the effects of LPA on action potentials and I_Ca,L_ are PTX-sensitive, suggesting LPA action requires a G_i_-type G protein. In conclusion, LPA prolongs APD and increases electrophysiological instability in isolated rabbit myocardial preparations by increasing I_Ca,L_ in a G_i_ protein-dependent manner.

## Introduction

Lysophosphatidic acid (LPA) is an intermediate molecule produced during phospholipid metabolism. A water-soluble glycerol phospholipid with a simple structure, LPA is secreted from numerous cell types, such as platelets, fibroblasts, and ovarian cancer cells [1], [2], and is present in very small amounts in human serum (with a concentration of 1–5 µM). It functions both as a component of the cell membrane and as an intracellular phospholipid signaling molecule [3] as an autocrine or paracrine mediator. Numerous and diverse biological processes are mediated by LPA, including cellular proliferation, cellular migration, differentiation, anti-apoptosis, actin cytoskeletal rearrangements, platelet aggregation, calcium mobilization, and neurotransmitter release [4]. LPA’s biological functions are mediated by at least six G protein-coupled receptors (GPCRs) referred to as LPA1–6 [5]–[8], which are widely distributed in the brain, heart, kidney, spleen and other organs. These receptors couple to multiple G proteins, notably G_12/13_, G_i_, G_q_, and possibly G_s_ [5]. LPA activates multiple signaling cascades, including phosphoinositide 3-kinase, phospholipase C, mitogen-activated protein kinase, Rho family GTPase, and adenylyl cyclase [9], [10]. Interestingly, as one of its roles as an extracellular mediator, LPA has also been proposed to serve as an endogenous activator of the nuclear peroxisome proliferator activated receptor gamma (PPARγ) [11], [12]. The assignment of specific biological functions to individual GPCR subtypes has been hampered by the overlapping expression of LPA receptors, their coupling to multiple G-proteins and their regulation of diverse signal transduction pathways. These characteristics result in pleiotropic responses in functional experiments, depending the cell type and the relative expression of LPA receptors [13].

LPA has important functions in the cardiovascular system, such as the induction of vascular smooth muscle contraction, the promotion of platelet aggregation, the stimulation of vascular smooth muscle cell and cardiac fibroblast proliferation, the promotion of cardiac hypertrophy, and the modulation of myocardial contractility [14]. Western blot and Northern blot analysis have indicated that the whole Endothelium Differentiation Gene (EDG)/LPA receptor family is expressed ubiquitously throughout the cardiovascular system. Thus, LPA may play a role in modulating cardiac function under physiological and/or pathological conditions. Accumulating evidence indicates that LPA plays an important role in regulating ion currents in multiple cell types [15]–[17]. However, the importance of LPA in regulating ion currents in myocardiocytes has not been studied. This work, using isolated myocardial preparations, examines the effects of LPA on action potential duration (APD) and membrane currents, and analyzes the possible underlying mechanisms. Overall, this study demonstrates that LPA is a key electrophysiological mediator in myocardiocytes.

## Materials and Methods

### Ethics Statement

All animal protocols in this study were approved by the Animal Care and Use Committee, Research Institute of Medicine, Shanghai Jiao Tong University, in accordance with National Institutes of Health guidelines and public law. All efforts were taken to minimize animal suffering.

### Cell Preparations

Male adult New Zealand White rabbits (1.5 to 2.5 kg) were used. Single ventricular myocytes were dissociated enzymatically as previously described [18]. Briefly, rabbits were heparinized (200 U/kg IV) and anesthetized with sodium pentobarbital (50 mg/kg IV). Hearts were excised via thoracotomy, and the aortas were rapidly cannulated. Isolated hearts were mounted on a Langendorff apparatus and were retrogradely perfused through the aorta with Ca^2+^-free Tyrode’s solution (135 mM NaCl, 5.4 mM KCl, 1 mM MgSO_4_, 0.33 mM NaH_2_PO_4_, 10 mM glucose, 10 mM HEPES, pH adjusted to 7.35 with NaOH) for 7 minutes. Samples were then treated with the same solution containing 0.5–0.8 mg/mL collagenase B (Worthington Chemical Co) and 0.06 mg/mL protease XIV (Sigma Chemical Co.) for 15–20 minutes, followed by storage solution for 5 minutes, at a rate of 3–4 mL/min. The ventricle was then cut into small pieces, pipetted, and filtered through 200 µm nylon mesh. The isolated myocytes were stored in KB solution (50 mM L-glutamic acid, 3 mM MgCl_2_, 20 mM taurine, 0.5 mM EGTA, 10 mM glucose, 10 mM HEPES, 20 mM KH_2_PO_4_, 70 mM KOH, 40 mM KCl, pH adjusted to 7.35 with KOH) at 4°C and were used for electrophysiological recordings 2–10 hours after isolation. Throughout the isolation procedure, all solutions were oxygenated with 100% O_2_ and solution temperatures were maintained at 37°C.

### Electrophysiological Recordings from Cell Preparations

A single-pipette whole-cell patch-clamp was applied to record action potentials and ion currents. The tip resistance of the glass pipette was 2.5 to 3.5 MΩ after being filled with an internal pipette solution. Action potentials were recorded in normal Tyrode’s solution (135 mM NaCl, 5.4 mM KCl, 1.8 mM CaCl_2_, 1 mM MgCl_2_, 0.33 mM NaH_2_PO_4_, 10 mM HEPES, 10 mM glucose, pH adjusted to 7.35 with NaOH). The pipette solution consisted of 120 mM KCl, 1 mM CaCl_2_, 5 mM MgCl_2_, 5 mM Na_2_ATP, 11 mM EGTA, 11 mM glucose, and 10 mM HEPES (pH adjusted to 7.35 with KOH). Action potentials were elicited by application of a 15-ms depolarizing pulse through the pipette and were recorded at frequencies of 0.25 to 4.0 Hz. Action potential duration (APD) was measured at 90% and 50% of repolarization (APD_90_ and APD_50_). Ion currents were normalized to cell capacitance and expressed as current densities (pA/pF) to control cell size variability. For L-type calcium current (I_Ca,L_) recordings, the bath solution was normal Tyrode’s solution and the pipette solution was 120 mM CsCl, 5 mM MgCl_2_, 5 mM Na_2_ATP, 11 mM EGTA, 11 mM glucose, and 10 mM HEPES (pH adjusted to 7.35 with CsOH). The cell membrane was clamped to a holding potential of −40 mV to inactivate Na^+^ and T-type calcium currents. I_Ca,L_ was elicited by a sequence of stimulation pulses. The standard voltage clamp protocol was a series of 300 ms voltage steps from −40 to +60 mV at a frequency of 0.25 Hz. The internal pipette solution for recording transient outward potassium current (I_to_) and delayed rectifier potassium current (I_K_) was composed of 60 mM KOH, 80 mM KCl, 40 mM aspartate, 5.0 mM HEPES, 10 mM EGTA, 5.0 mM MgATP, 5.0 mM sodium creatine phosphate, and 0.65 mM CaCl_2_ (pH adjusted to 7.2 with NaOH). For I_to_ recordings, the bath solution was normal Tyrode’s solution with nisoldipine (3 µM) added to block I_Ca,L_. I_to_ was elicited from the holding potential of −80 mV with a subsequent 20-ms pre-pulse of −50 mV and by a series of 300 ms voltage steps from −40 to +50 mV at a frequency of 0.1 Hz. To measure I_K_, cells were superfused with Tyrode’s solution mixed with CdCl_2_ (100 µM), BaCl_2_ (100 µM), tetrodotoxin (50 µM), and 4-aminopyridine (1 mM) to eliminate I_Ca,L_, inward-rectifier potassium current (I_K1_), Na^+^ current and I_to_ respectively. I_K_ was elicited from the holding potential of −50 mV and by a series of 3000 ms voltage steps from −40 to +50 mV at a frequency of 0.1 Hz. Optimized compensation is essential for eliminating linear components from the current signal when investigating I_Ca,L_ and I_K_. When recording action potential, I_to_ and I_K_, data were collected before and after 10-min perfusion with LPA in the same cell. When recording I_Ca,L_, cells were randomly divided into two groups (group Control and group LPA) and only the initial recording data were collected. Voltage and current signals were filtered at 2 kHz and stored on a personal computer with PatchMaster software (EPC 10, HEKA Instruments) for analysis.

### Arterially Perfused Rabbit Left Ventricular Wedge Preparations

The methodology for preparation of the arterially perfused ventricular wedge was similar to that previously described [19]. Briefly, 20 rabbits were anaesthetized with sodium pentobarbital (50 mg/kg IV) and anticoagulated with heparin (200 U/kg IV). The heart was excised and submerged in cold (4°C) cardioplegic solution (109 mM NaCl, 24 mM KCl, 20 mM NaHCO_3_, 0.9 mM NaH_2_PO_4_, 0.5 mM MgSO_4_, 1.8 mM CaCl_2,_ 5.5 mM glucose, buffered with 95% O_2_ and 5% CO_2_, with pH adjusted to 7.35 with NaOH). The left circumflex branch of the coronary artery was cannulated and perfused with the same cardioplegic solution. Unperfused areas of the left ventricle were easily identified by their reddish appearance due to the presence of unflushed erythrocytes, and were removed. The cannulated preparation was then placed in a small heated tissue bath and arterially perfused with Tyrode’s solution (129 mM NaCl, 4 mM KCl, 0.9 mM NaH_2_PO_4_, 20 mM NaHCO_3_, 1.8 mM CaCl_2_, 0.5 mM MgSO_4_, 5.5 mM glucose, buffered with 95% O_2_ and 5% CO_2_, and maintained at 35.7±0.2°C). The perfusate was delivered to the artery by constant flow with a roller pump. Perfusion pressure was monitored continuously with a pressure transducer and maintained at 35 to 45 mmHg.

### Electrophysiological Recordings from the Wedge Preparations

The endocardial surface of the preparations was stimulated with bipolar silver electrodes, insulated except for the tips. A transmural pseudo-electrocardiogram (ECG) of the wedge was recorded with a pair of silver/silver chloride electrodes positioned in the bath at opposite sites of the wedge and along the same vector as the transmembrane recordings. Monophasic action potentials were recorded from the endocardium using floating glass microelectrodes, with a resistance of 10–20 MΩ after being filled with potassium chloride solution (2.7 M). The impalement of microelectrodes was maintained approximately on the axis of the transmural ECG recording electrodes. Induced ventricular tachycardia (VT) was determined by pacing the apex endocardium of the arterially perfused ventricular wedge preparations with trains of eight conditioning current pulses (S_1_), delivered at predetermined rates (1.0 Hz), with each train being followed by a premature pulse (S_2_) of the same magnitude. The S_1_–S_2_ interval was progressively shortened in 10 ms steps. Induced VT was defined as over 3 continuous premature ventricular beats induced by S_2_.

**Figure 1:**
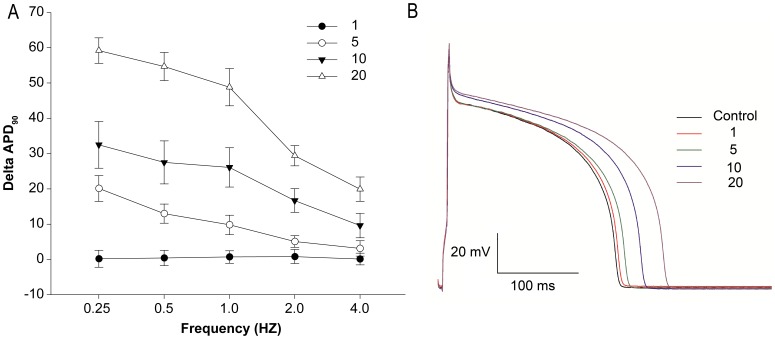
Concentration and frequency-dependent prolongation of action potential duration (APD) induced by LPA in isolated rabbit ventricular myocytes. (A) Action potentials were recorded at different frequencies from 0.25 to 4.0 Hz before and after perfusion with various concentrations of LPA. Closed circles: 1 µM, open circles: 5 µM, closed triangles: 10 µM, open triangles: 20 µM. Delta APD_90_ is defined as the difference of APD_90_ recorded before and after LPA addition at a constant frequency (n = 10). (B) Representative traces of action potential recorded at the frequency of 1.0 Hz in cardiac myocytes without (Control) and with different concentrations of LPA (1, 5, 10, 20 µM).

### Solution and Drug Preparation

The LPA (1-Oleoyl-sn-Glycero-3-Phosphate), protease XIV, MgATP, CsCl, CsOH, Na_2_ATP, tetrodotoxin, 4-aminopyridine and HEPES used in this study were obtained from Sigma Chemical Co. Collagenase B was purchased from Worthington Chemical Co. All other chemicals were obtained from China National Medicines Corporation Ltd. A stock solution (0.1 mg/mL) of LPA was prepared in calcium- and magnesium-free buffers and subsequently diluted in extracellular bath solution to the desired concentration.

**Figure 2:**
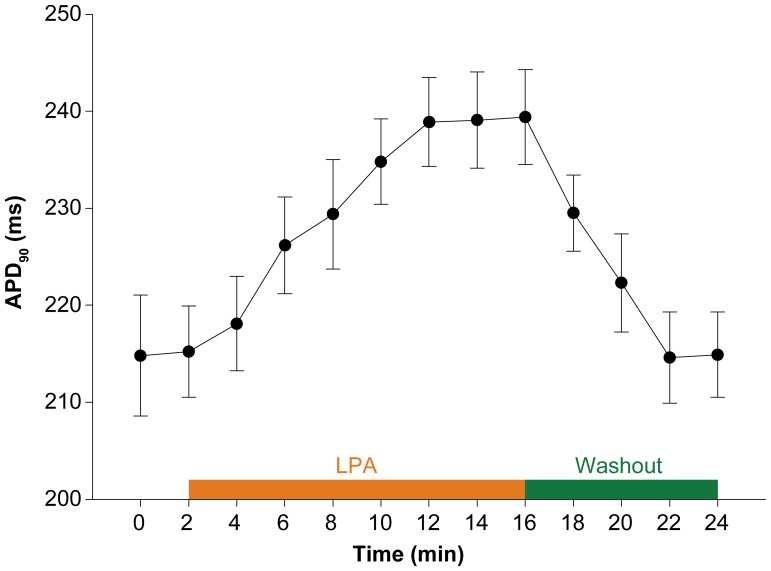
Time course of changes in action potential duration (APD) at 1.0 Hz in isolated rabbit ventricular myocytes after addition of LPA (10 µM). (n = 10, ANOVA, P<0.01).

### Statistics

All continuous data are expressed as mean±SDM. Statistical significance was determined with the sample-paired Student’s t-test for paired observations: APD_90_, QT interval, Tpeak-end, and MAPD_90_ were recorded in conditions before and after LPA perfusion. Unpaired group comparisons of I_Ca,L_ (ex. groups without LPA vs groups with LPA) were made with the independent Student’s t-test. These two statistical approaches were used because the spontaneous rundown of I_Ca,L_ prevents recording paired observations before and after LPA perfusion. A two-way ANOVA test was performed to compare current densities as a function of both membrane potential and LPA treatment. Proportions of induced VT occurrences by S_1_S_2_ stimulation before and after LPA perfusion were compared using the Fisher exact test. Differences were considered significant at P<0.05.

Data for voltage-dependence of activation and inactivation of I_Ca,L_ were fitted to the Boltzmann equation: Y = 1/{1+exp[−(V_m_−V_1/2_)/K], where Vm is the membrane potential, V_1/2_ is the half-activation or half-deactivation potential, and K is the inverse slope factor. For activation-Vm curves, Y represents the relative conductance (G/G_max_). For inactivation-Vm curves, Y represented the relative current (I/I_max_).

**Table 1:** Effect of LPA (10 µM) on action parameters recorded in rabbit ventricular myocytes at the frequency of 1 Hz.

	Control	LPA	Washout
RMP (mV)	−85.50±1.43	−85.6±1.71	−85.8±2.04
V_max_ (V/s)	153.40±1.90	153.90±2.13	153.60±1.84
APA (mV)	120.40±3.41	120.80±3.16	119.70±4.52
OS (mV)	26.20±1.48	26.00±1.49	26.60±1.35
APD_20_ (ms)	122.60±2.88	122.20±2.15	121.60±3.10
APD_50_ (ms)	175.50±4.43	200.60±3.66*	174.90±4.23
APD_90_ (ms)	215.80±9.37	241.90±6.66*	213.60±9.00

APA, action potential amplitude. RMP, resting membrane potential. OS, overshoot. APD_90_, 90% of action potential duration. APD_50_, 50% of action potential duration. APD_20_, 20% of action potential duration. *P<0.01 in paired t-test, compared with the baseline (n = 10).

**Figure 3:**
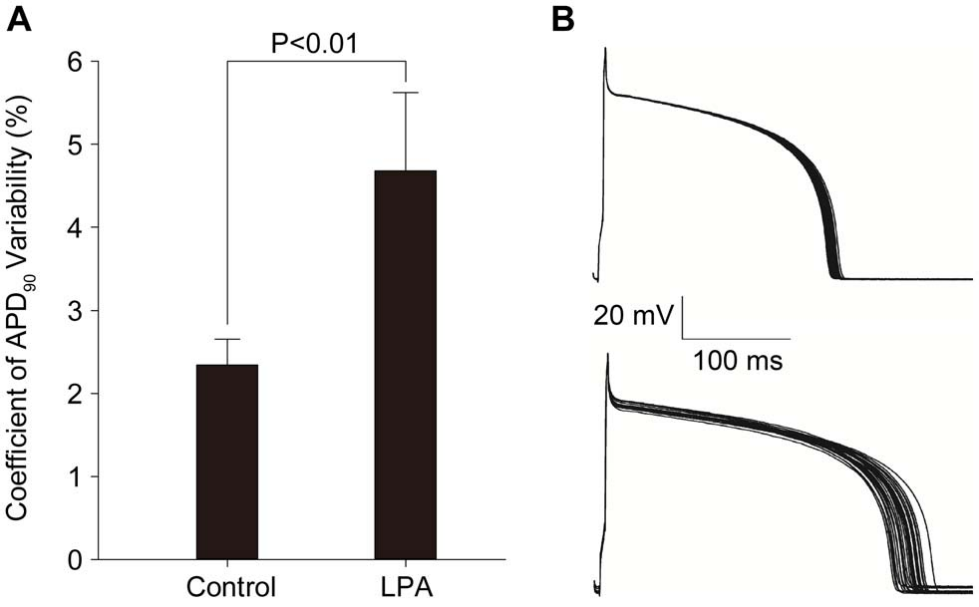
LPA-induced increase in action potential duration (APD) variability in isolated rabbit ventricular myocytes. (A) Comparison of APD_90_ variability subsequently recorded at 1.0 Hz before and after LPA treatment (10 µM). *P<0.01 in paired t-test (n = 10). (B) Representative action potentials recorded from 30 consecutive beats in one adult rabbit ventricular myocyte without (upper panel) and with (lower panel) LPA treatment. Coefficient of APD_90_ variability = (SD/mean APD_90_)×100%.

## Results

### LPA Prolongs APD in Isolated Adult Rabbit Ventricular Myocytes

To test whether LPA mediates action potential duration (APD) in singly isolated adult rabbit ventricular myocardiocytes, APD before and after LPA treatment was examined. The data reveal that LPA prolongs APD in a concentration- and frequency-dependent manner (Fig. 1). ΔAPD_90_ is the difference between APD recorded at a constant frequency before and after LPA addition (ΔAPD_90_ = APD_90_ pre-LPA – APD_90_ post-LPA). Four LPA concentrations were tested: 1, 5, 10, and 20 µM. A dose of 1 µM did not alter APD_90_ at any frequency. A prolongation of APD appeared at 5 µM and had the greatest magnitude at 20 µM LPA. A mid-level concentration (10 µM) was selected for subsequent experiments.

**Figure 4:**
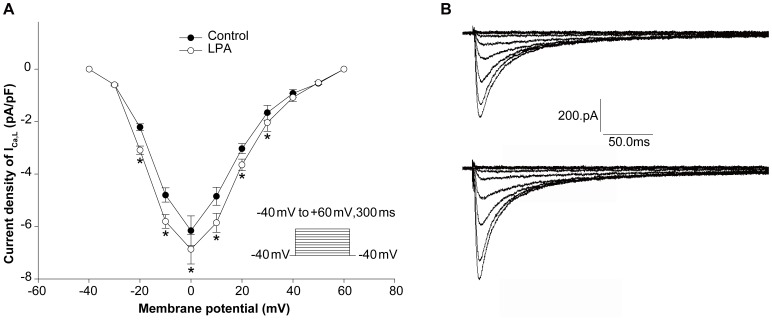
Effect of LPA on current-voltage relation of L-type calcium current (I_Ca,L_) in isolated rabbit ventricular myocytes. (A) Current amplitudes corrected for cell size in the presence (open circles) and absence (solid circles) of LPA (10 µM) plotted against the test potentials. Error bars are SDM. *P<0.01, two-way ANOVA followed by the Bonferroni test, comparing peak current density of I_Ca,L_ at the same potential recorded in myocytes without LPA (n = 10). (B) Representative traces of I_Ca,L_ in cardiac myocytes without (upper panel) and with (lower panel) LPA.

Next, a time course of LPA’s effect on APD prolongation in rabbit ventricular myocytes at 1.0 Hz was performed (Fig. 2). APD gradually increased after LPA addition, with the prolongation of APD appearing at 2 min and peaking after 10 min of treatment. The prolonged APD induced by LPA was readily reversible after washing out. Therefore, we used a LPA treatment time of 10 minutes for subsequent experiments.

**Figure 5:**
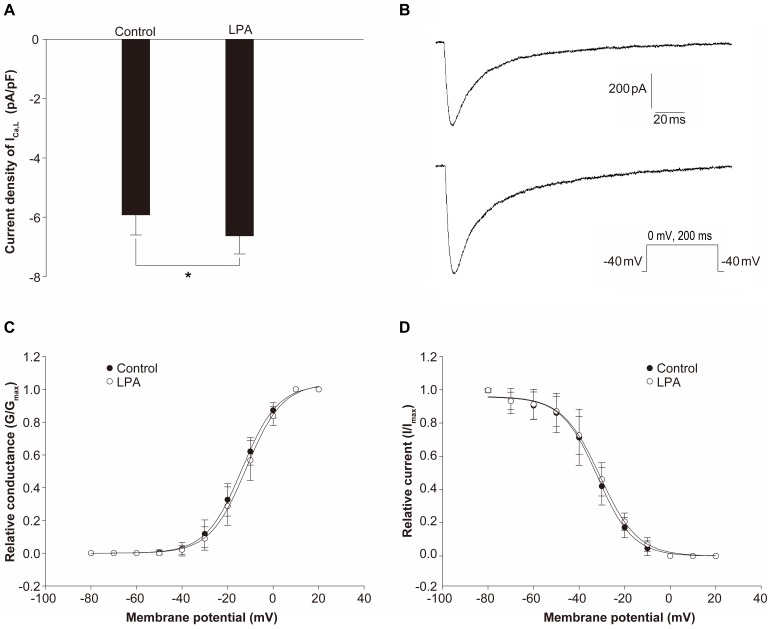
LPA increases L-type calcium current (I_Ca,L_) density without altering activation or inactivation properties. (A) Average current densities of I_Ca,L_ in the absence and presence of LPA (10 µM). The isolated rabbit ventricular myocytes were kept at a holding potential of −40 mV and depolarized to 0 mV for 200 ms. *P<0.05 compared with the control in an Independent Samples t-Test (n = 10). (B) Representative I_Ca,L_ in the control condition (upper panel) and LPA treatment (lower panel). Voltage dependence of I_Ca,L_ activation (C) and inactivation (D) in the control condition (closed circles, n = 10) and in the presence of LPA (open circles, n = 10).

Treatment with LPA prolongs both APD_90_ and APD_50_ in ventricular myocytes at the frequency of 1 Hz. APD_90_ increased from 215.80±9.37 ms to 241.90±6.66 ms (P<0.01), while APD_50_ increased from 175.50±4.43 ms to 200.60±3.66 ms (P<0.01). Notably, other action potential parameters, such as resting membrane potential (RMP), maximal rate of depolarization (V_max_), overshoot, and action potential amplitude (APA) did not change after LPA addition and washout (Table 1).

Beat-to-beat variation in APD exists in single ventricular myocytes paced at a constant rate [20]. It is an index positively associated with electrophysiological instability in myocardiocytes and contributes to arrythmogenesis [21]. To examine APD_90_ variability, the coefficient of variability (CV) was calculated by dividing the standard deviation of 30 consecutive APD_90_ at 1.0 Hz by their mean (CV = SD/mean APD_90_×100%). The application of LPA increased the CV from 2.34%±0.31 to 4.68%±0.94 (n = 10, P<0.01, Fig. 3A). These results demonstrate that LPA significantly increases APD_90_ variability. This change is also reflected in traces of representative action potentials recorded from 30 consecutive beats of one adult rabbit ventricular myocyte with and without LPA (Fig. 3B–C).

**Table 2:** Parameters of voltage-dependent I_Ca,L_ activation and deactivation in adult rabbit cardiomyocytes.

	Activation		Deactivation	
	V_1/2_ (mV)	K (mV)	V_1/2_ (mV)	K (mV)
Control (n = 10)	−13.50±2.30	7.15±1.38	−33.00±4.60	−8.10±0.83
LPA (n = 10)	−12.39±3.70	7.57±0.77	−31.10±3.28	−8.28±0.89
P value	0.434	0.421	0.302	0.815

### Effects of LPA on I_Ca,L_ in Isolated Adult Rabbit Ventricular Myocardiocytes

To determine whether I_Ca,L_ can explain APD prolongation, this current was examined. Voltage-clamp steps were applied for 300 ms from a holding potential of −40 mV to different depolarizing levels up to +60 mV at 0.25 Hz. Current-voltage (I–V) curves of the peak I_Ca,L_ densities indicate that I_Ca,L_ densities in myocytes with LPA treatment are higher than those in untreated myocytes from −20 mV to +30 mV (Fig. 4A). Representative traces of I_Ca,L_ in cardiac myocytes with (lower panel) and without (upper panel) LPA treatment are shown in Figure 4B.

**Figure 6:**
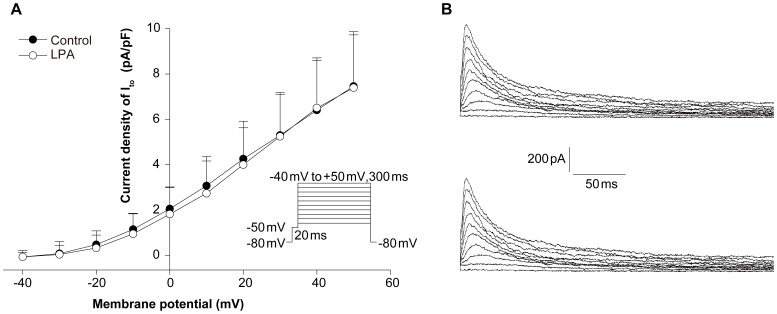
LPA does not affect the current-voltage relation of transient outward potassium current (I_to_) in isolated rabbit ventricular myocytes. (A) Current amplitudes corrected for cell size observed in the presence (open circles) and in the absence (solid circles) of LPA were plotted against the test potentials. Error bars are SEM, n = 8. (B) Representative traces of I_to_ in cardiac myocytes without (upper panel) and with (lower panel) LPA.

**Figure 7:**
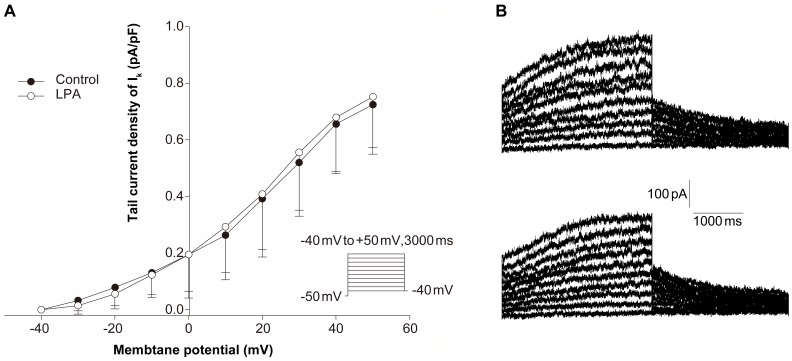
LPA does not affect the current-voltage relation of delayed rectifier potassium current (I_K_) in isolated rabbit ventricular myocytes. (A) Current amplitudes corrected for cell size observed in the presence (open circles) and in the absence (solid circles) of LPA plotted against the test potentials. Error bars are SEM. No significant difference was observed, n = 7. (B) Representative traces of I_K_ in cardiac myocytes without (upper panel) and with (lower panel) LPA treatment.

To verify that LPA treatment increases I_Ca,L_, the single-wave protocol was used to record single I_Ca,L_ traces of at a holding potential of −40 mV and a test potential of 0 mV for 200 ms. LPA treatment significantly increase I_Ca,L_ density from −5.92±0.68 pA/pF to −6.63±0.61 pA/pF (n = 10, *P*<0.05, Fig. 5A). Representative current traces of I_Ca,L_ in the absence (upper panel) and presence (lower panel) of LPA are shown in Fig. 5B. Though G_max_ (LPA vs Control, 17.03±1.91 vs 10.04±3.57 pS, *P*<0.05) was increased by LPA, it did not alter the activation curve of I_Ca,L_ (Fig. 5C), with no change in midpoint potential (V_1/2_) nor the slope factor (K) (Table 2). Inactivation properties were also unchanged with LPA treatment (Fig. 5D), with no change in V_1/2_ nor K (Table 2), regardless of an increased I_max_ (LPA vs Control, 6.74±0.51 vs 5.92±0.86 pA/pF, *P*<0.05). The time course of the run-down of *I*_Ca,L_ was not altered by LPA either (Data were showed in Figure S1).

**Figure 8:**
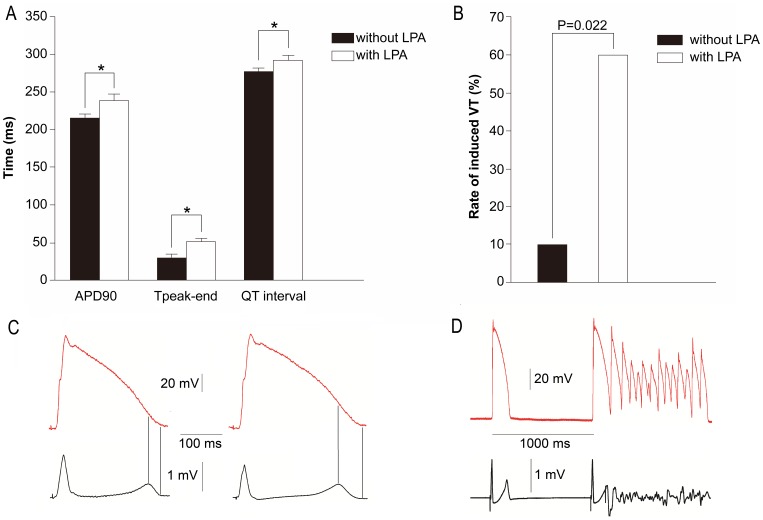
LPA treatment increases electrophysiological instability in the heart. LPA prolongs 90% of action potential duration (APD_90_), Tpeak-end, and QT interval (A) and increased the incidence of ventricular tachycardia (B) induced by S_1_S_2_ stimulation in the arterially perfused rabbit left ventricular wedge preparations, n = 10. (C) Representative monophasic action potential in the endocardium and the transmural ECG before (left) and after (right) LPA treatment. (D) Representative ventricular tachycardia induced by S_1_S_2_ stimulation in the presence of LPA.

### LPA has no Effect on I_to_ and I_K_ Ventricular Repolarizing Currents

Three main ion currents, I_Ca,L_, I_to_, and I_K_, play a key role in ventricular repolarization. Although increased I_Ca,L_ could be a plausible explanation for APD prolongation, it was unclear whether LPA also had effects on other ventricular repolarizing currents, namely I_to_ and I_K_. An examination of these currents indicates that LPA does not alter the I–V curves of neither I_to_ nor I_K_ (Fig. 6 and 7).

**Figure 9:**
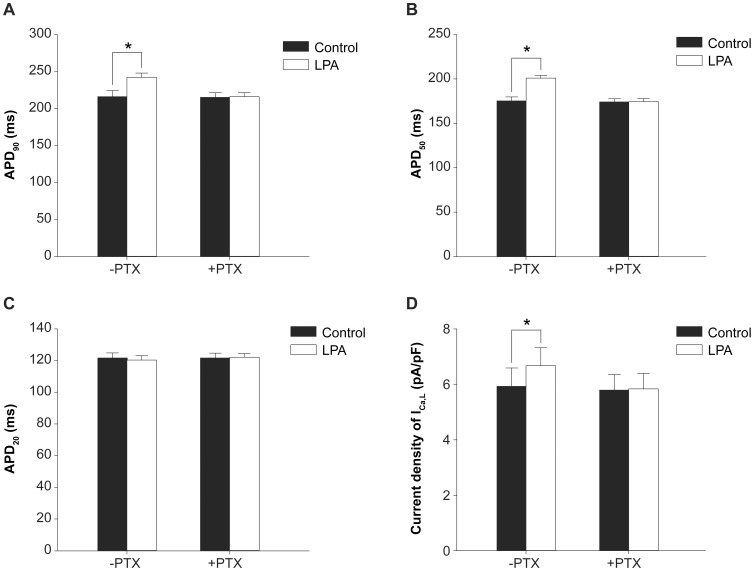
The effects of LPA on action potentials and I_Ca,L_ are abolished by pertussis toxin (PTX). LPA increases APD_90_ (A), APD_50_?(B)?and I_Ca,L_ (D) above basal values, which does not occur after PTX pretreatment, * P<0.05, comparing the LPA treated sample with the control in the absence or presence of PTX), n = 10. I_Ca,L_ was recorded with the single-wave protocol at a holding potential of −40 mV and a test potential of 0 mV for 200 ms. (C) LPA has no effect on APD_20_ without or with PTX pretreatment (n = 10).

### Impact of LPA on the Electrical Stability of Rabbit Left Ventricular Wedge Preparations

In order to confirm the electrophysiological effects of LPA on the myocardium, in vitro experiments were performed with arterially perfused rabbit left ventricular wedge preparations. After the addition of LPA (10 µM), monophasic action potential duration (MAPD90) was prolonged from 215.01±4.85 ms to 238.64±7.46 ms (P<0.01, Fig. 8A). In addition, an increase of QT interval from 276.47±4.53 ms to 291.40±6.49 ms (P<0.01) occurred upon treatment (Fig. 8A). Tpeak-end is defined as the interval between the peak and the end of the T wave, and has been previously verified as a reliable measure of transmural dispersion of repolarization, which may reflect arrhythmic risk [22], [23]. Tpeak-end increased with LPA treatment from 28.83±5.48 ms to 51.12±4.53 ms (P<0.01, Fig. 8A). In summary, MAPD, QT interval and Tpeak-end were all prolonged after LPA perfusion (Fig. 8C).

Further, the incidence of ventricular tachycardia (VT) induced by S_1_S_2_ stimulation was determined. Recordings were made in one of the ten myocytes before the addition of LPA and six of the ten myocytes following treatment. LPA significantly increased induced VT (Fig. 8B). Representative examples of VT induced by S_1_S_2_ stimulation and detected by simultaneously recording monophasic action potentials in the endocardium and the transmural ECG are shown in Fig. 8D.

### PTX Abolishes LPA-induced Effects on Action Potentials and I_Ca,L_

Previously, it has been observed that blocking G_i_ protein function with pertussis toxin (PTX) abolishes LPA-dependent modulation of myocardial contractility [3]. To test whether LPA’s effects on action potentials and I_Ca,L_ are dependent on G_i_ proteins, myocardiocytes were treated with PTX (Fig. 9). Compared to basal values, LPA treatment increases APD_90_ by 12% (Fig. 9A), APD_50_ by 15% (Fig. 9B), and I_Ca,L_ by 13% (Fig. 9C). Pretreatment of myocardiocytes with PTX (50 ng/mL) completely abolishes the LPA-induced prolongation of APD_90_ (Figure 9A) and APD_50_ (Figure 9B) and increase in I_Ca,L_ (Figure 9C). PTX treatment alone did not alter basal values. These results indicate that the effects of LPA on action potentials and I_Ca,L_ are PTX-sensitive, demonstrating the involvement of G_i_ protein in LPA signaling.

## Discussion

Lysophosphatidic acid (LPA) is an intermediate metabolite of phospholipid biosynthesis and functions as an intercellular lipid messenger [24]. Although LPA is widely reported to modulate multiple ion currents in some cell types, little was known about its electrophysiological effects on cardiac myocytes.

LPA has multiple activities in the cardiovascular system. It has been shown that LPA is involved in the processes of artherosclerosis and thrombogenesis [25], [26]. LPA has also been found to regulate blood vessel tone [27], to have positive inotropic effects in the heart in rats [28], and to induce a hypertrophic response in cultured neonatal myocardiocytes through various signaling pathways [29]–[31]. However, prior to this study, there had been no investigation on the potential actions of LPA on electrophysiological regulation of ion currents and APD in cardiac myocytes. In other cell types, it has been verified that LPA is involved in numerous electrophysiological activities. For example, LPA opens a P-type Ca^2+^ channel in human erythrocytes [15]. Chang and colleagues also demonstrated that LPA increases intracellular Ca^2+^ through endogenous LPA receptors in C6 glioma and L2071 fibroblasts [32]. In vascular smooth muscle cells, LPA has been found to induce Ca^2+^ mobilization, which involves not only extracellular Ca^2+^ entry through the sarcolemma Na^+^-Ca^2+^ exchanger, Na^+^-H^+^ exchanger and store-operated Ca^2+^ channels, but also Ca^2+^ release from ryanodine-sensitive and InsP(3)-sensitive intracellular Ca^2+^ pools [33], [34]. In addition, LPA has been shown to activate Cl^−^ current in cultured corneal keratocytes in a dose-dependent manner, which results in subsequent depolarization of the cells [16]. Furthermore, LPA can modulate potassium channels in neuronal cells [17], [35]. Together, these observations suggest that LPA plays an important electrophysiological role in multiple cellular processes. It was reasonable to hypothesize, therefore, that LPA might also function in cardiac myocytes as a regulator of cardiac electrophysiological activity.

This study provides functional evidence that LPA is a modulator of cardiac electrophysiological stability. LPA prolongs APD and causes changes in electrophysiological properties that are conducive to arrhythmia. The increase of I_Ca,L_ densities is associated with prolonged APD at a physiological cycle length as well as prolongation of QT interval and Tpeak-end in ECG. Although low concentrations of LPA do not affect APD in isolated rabbit ventricular myocytes, high concentrations (5–20 µmol/L) produce a significant increase in the APD. LPA can thus be considered as an endogeneous pro-arrhythmia factor if it reaches high levels in the plasma. This newly discovered action of LPA on the heart could be of pathophysiological importance in conditions like acute myocardial infarction, in which LPA is massively released by activated platelets.

Given the pronounced effects of LPA on APD prolongation, which ion channels are responsible for this action was an essential question to address. De Jong and colleagues have demonstrated that products secreted from activated human platelets induce changes in I_Ca,L_ and [Ca^2+^]_i_, which result in action potential prolongation and the occurrence of EAD and DAD in rabbit myocytes [36]. Interestingly, although products of activated platelets consist of organic substrates and more than 2000 proteins, LPA is an important component [37]. I_Ca,L_ plays an important role in the plateau phase of the action potential and is one of the key ion currents determining APD. Experimental results indicate that LPA treatment increases the current density of I_Ca,L_. The increase of the inward I_Ca,L_ may therefore explain APD prolongation and variation. That LPA affects cellular electrophysiological properties, thereby modulating vulnerability to VT, supports the idea that LPA exerts pro-arrhythmic effects. Our data are mainly consistent with the previous study of Xu et al [28], who found that LPA had the positive inotropic effect in rat heart and such action was abolished by the antagonist of L-type Ca^2+^ channel (Verapamil). They also demonstrated LPA (1–30 µM) failed to alter the basal intracellular concentration of free Ca^2+^ in freshly isolated rat myocardiocyte [28]. Nonetheless, our study indicates LPA is shown to increase I_Ca,L_ in rabbit myocardiocytes, which may cause Ca^2+^ influx and increase [Ca^2+^]_i_. The observed inability of LPA to increase [Ca^2+^]_i_ in Xu’s study may be due to [Ca^2+^]_i_ measurement in quiescent myocardiocytes only, without using electrically-stimulated myocardiocyte preparations.

LPA is reported to be present in serum at concentrations ranging from 1–5 µM under normal physiological conditions [38], and can reach up to 20 µmol/L in patients following acute myocardial infarction (AMI). During this condition, in response to injury and inflammation, LPA is massively released by thrombin-activated platelets [39]. Serum LPA levels can be increased more than two-fold 8 h after the onset of AMI, with maximum levels occurring at 48–72 h following onset. LPA concentration remains higher than basal levels 7 days after injury [40]. Based on these previous studies and the current work, LPA likely represents a crucial link between AMI and the development of malignant arrhythmia. After injury, the consequential impairments in repolarization may predispose the ischemic heart to lethal ventricular arrhythmias. Abnormally high levels of LPA, together with temporally and spatially selective expression of LPA receptor subtypes in disease conditions, may also account for the diverse bioactivities of LPA. This molecule may exert potentially deleterious electrophysiological effects on myocardiocytes under certain disease conditions, including not only AMI, but also heart failure and hypertrophic myopathy. Since LPA is released from activated platelets, it could be considered as a marker for predicting the incidence and severity of malignant arrhythmias after acute injury. LPA may be regarded as an endogenous pro-arrhythmic factor in some pathophysiological conditions. This work implies that inactive LPA analogues that compete for receptor binding may be an effective new approach to preventing arrhythmia.

Limitations of the present study include that isolated myocardiocytes may not represent the physiological response to LPA of in vivo myocardiocytes. In addition, the identification of individual LPA receptors that mediate specific ion currents is problematic for single myocardiocytes, in which there may be multiple LPA receptor subtypes, with each receptor subtype potentially signaling via multiple G proteins. Several lines of evidence indicate that the effects of LPA on cardiac myocytes are mediated through receptors coupling to various G proteins, including G_i_, G_q_, and G_12/13_. However, specific antagonists for each LPA receptor subtype, which would allow the dissection of subtype-specific functions, are not currently available. For these reasons, this study could not identify the LPA receptor subtypes linked with the specific pathways underlying APD prolongation and the increase in I_Ca,L_. Nevertheless, the effects of LPA on action potentials and I_Ca,L_ are PTX-sensitive, indicating the involvement of G_i_ proteins in LPA signaling. In the future, animals in which specific LPA-receptors are knocked-out will be required to determine which LPA receptor subtypes are linked to modulation of particular ion channels in cardiac myocytes.

In summary, these results suggest that the LPA-mediated APD prolongation and increase of electrophysiological instability in adult rabbit ventricular myocardium are dependent on an increase in I_Ca,L_. Importantly, this work provides evidence for a role of LPA in the process of arrhythmia, and implies that the blockade of LPA receptors could be a new approach for reducing the risk of ventricular arrhythmias under pathological conditions.

## Supporting Information

Figure S1 **The time course of the run-down of** ***I*** **_Ca,L_ in conditions without and with LPA bath.** *I*_Ca,L_ was recorded by the single-wave protocol (at a holding potential of −40 mV and a test potential of 0 mV for 200 ms), *n = *8.
(TIF)
