# Supplementary figures and images for: Lysophosphatidic Acid Increases the Electrophysiological Instability of Adult Rabbit Ventricular Myocardium by Augmenting L-Type Calcium Current
Source: PLoS One. 2012 Sep 21;7(9):e45862. doi: 10.1371/journal.pone.0045862 (PMC3448719; doi:10.1371/journal.pone.0045862)

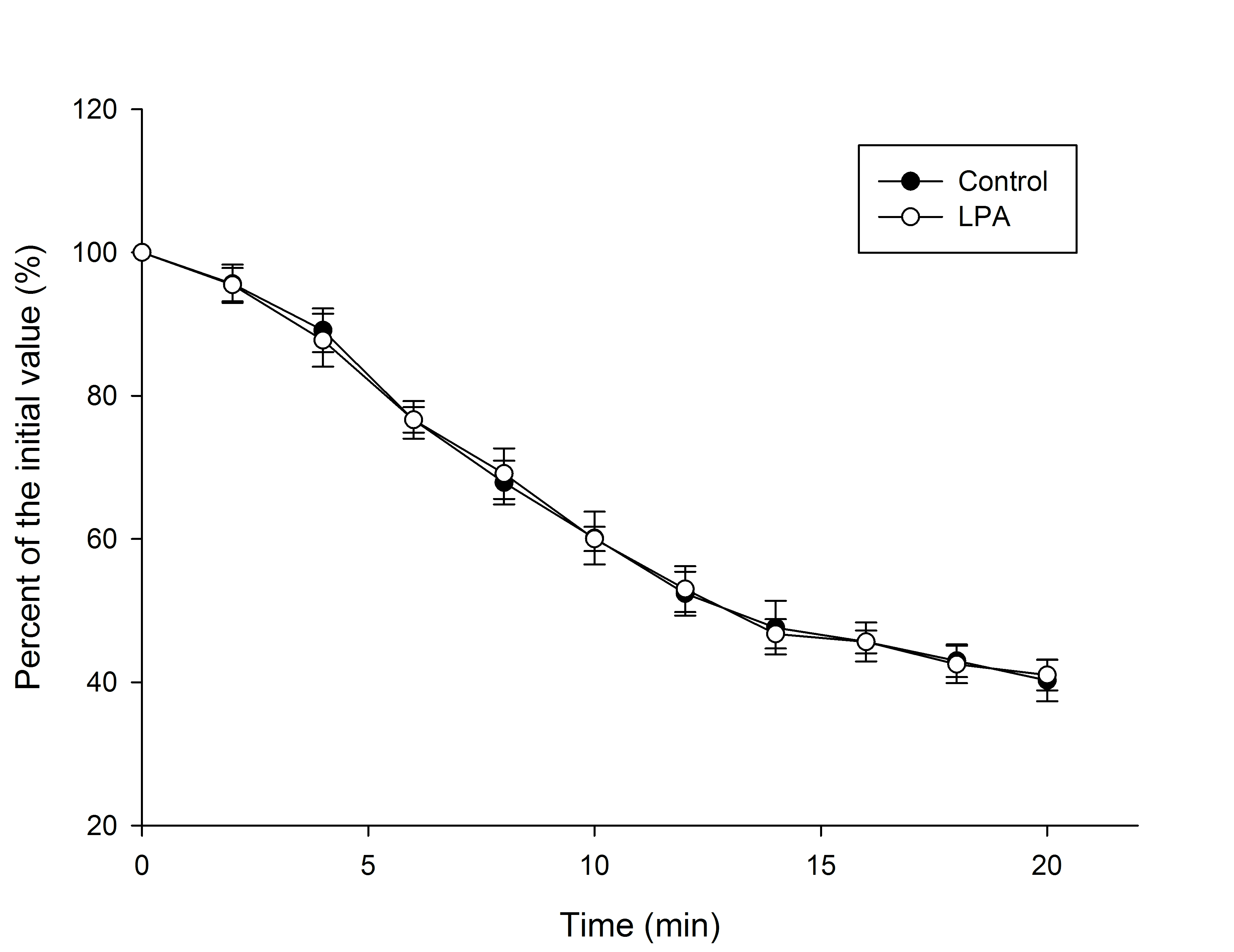

Supplement: Figure S1 — The time course of the run-down of I Ca,L in conditions without and with LPA bath. I Ca,L was recorded by the single-wave protocol (at a holding potential of −40 mV and a test potential of 0 mV for 200 ms), n = 8. (TIF) [file pone.0045862.s001.tif]
